# A Model for ERD2 Function in Higher Plants

**DOI:** 10.3389/fpls.2020.00343

**Published:** 2020-03-25

**Authors:** David G. Robinson, Fernando Aniento

**Affiliations:** ^1^Centre for Organismal Studies, University of Heidelberg, Heidelberg, Germany; ^2^Departamento de Bioquimica y Biologia Molecular, Estructura de Recerca Interdisciplinar en Biotecnologia i Biomedicina (ERI BIOTECMED), University of Valencia, Valencia, Spain

**Keywords:** *cis*-Golgi, COPI-vesicle, COPII-vesicle, ERD2/KDEL receptor, K(H)DEL ligand, secretory unit

## Abstract

ER lumenal proteins have a K(H)DEL motif at their C-terminus. This is recognized by the ERD2 receptor (KDEL receptor in animals), which localizes to the Golgi apparatus and serves to capture escaped ER lumenal proteins. ERD2-ligand complexes are then transported back to the ER via COPI coated vesicles. The neutral pH of the ER causes the ligands to dissociate with the receptor being returned to the Golgi. According to this generally accepted scenario, ERD2 cycles between the ER and the Golgi, although it has been found to have a predominant Golgi localization. In this short article, we present a model for the functioning of ERD2 receptors in higher plants that explains why it is difficult to detect fluorescently tagged ERD2 proteins in the ER. The model assumes that the residence time for ERD2 in the ER is very brief and restricted to a specific domain of the ER. This is the small disc of ER immediately subjacent to the first *cis*-cisterna of the Golgi stack, representing specialized ER export and import sites and therefore constituting part of what is known as the “secretory unit”, a mobile aggregate of ER domain plus Golgi stack. ERD2 molecules in the ER domain of the secretory unit may be small in number, transient and optically difficult to differentiate from the larger population of ERD2 molecules in the overlying Golgi stack in the confocal microscope.

## Introduction

ERD2 in yeast and plants, or KDELR in mammalian cells, is a multi-spanning transmembrane receptor which is responsible for the retrieval of ER-luminal proteins from the Golgi apparatus (Capitani and Sallese, 2009). Such proteins have typically a K(H)DEL motif at their C-terminus. It is generally accepted that KDELR-ligand complexes in animal cells, interact with ARF1 and p24 proteins causing them to be incorporated into nascent COPI vesicles at the periphery of Golgi cisternae (Pastor-Cantizano et al., 2016; Arakel and Schwappach, 2018; Béthune and Wieland, 2018; Aniento et al., 2019). KDELR-ligand dissociation occurs after fusion of the COPI vesicles with the ER, and the receptors are then presumed to be returned to the Golgi apparatus via COPII vesicles (Gomez-Navarro and Miller, 2016). KDELR therefore cycles between the Golgi apparatus and the ER, and it has been detected in animal cells by immunological methods in the *cis*-Golgi, in COPI vesicles, in the ERGIC (ER-Golgi-Intermediate compartment) and partially also in the ER (Tang et al., 1993; Griffiths et al., 1994; Orci et al., 1997). However, under steady state conditions, the highest concentration of KDELR is visualized in the *cis-*Golgi (Griffiths et al., 1994). Its presence in the ER is especially notable under conditions in which KDELR has been overexpressed (Tang et al., 1993), or when a higher amount of KDEL ligands need to be transported from the Golgi to the ER, either following KDEL ligand overexpression (Lewis and Pelham, 1992; Bräuer et al., 2019) or when a wave of KDEL ligand (e.g., cholera toxin) arrives at the Golgi (Majoul et al., 1998).

The molecular basis for ligand binding and KDELR cycling between the ER and Golgi apparatus has recently been precisely defined for chicken KDELR and is pH dependent, but calcium independent (Bräuer et al., 2019). Optimal ligand binding occurs at a pH below 6 (Wilson et al., 1993), although the measured pH in the *cis*-Golgi of mammalian cells lies around 6.5 (Lee et al., 2019). Dissociation occurs in the ER which has a pH of 7.2-7.4 (Wu et al., 2000). At neutral pH, several acidic residues (Asp^87^, Glu^143^, Glu^145^) may form part of a diacidic COPII-binding ER exit motif, but the acid pH in the *cis*-Golgi causes a conformational change in KDELR structure thereby revealing arginine residues in transmembrane domains 1, 2 and 6 for KDEL ligand binding, and also exposing a classic COPI-binding dilysine cluster on the surface of the transmembrane domain 7 for retrograde traffic (Bräuer et al., 2019). Consistently, KDELR mutants with impaired KDEL binding failed to relocalize to the ER upon KDEL ligand overexpression, as happens with KDELR mutants in the lysine residues responsible for COPI binding (Bräuer et al., 2019). Strikingly, most of the residues involved in KDEL binding, as well as those involved in COPI or COPII binding, are highly conserved in *Arabidopsis* ERD2 proteins.

### Plant ERD2 Proteins and Their Subcellular Localization

The Arabidopsis genome has seven ERD2-like proteins that may be grouped into two classes (Xu and Liu, 2012), but in contrast to studies on animal cells, ERD2 in plants has only been localized hitherto through GFP-technology. Since ERD2-C(Y)FP appeared to be functionally active in animal cells (Majoul et al., 2001), the first ERD2 localization data in plant cells was also gained using C-terminally tagged fusion proteins. These studies showed ERD2 to be primarily found in Golgi stacks (Brandizzi et al., 2002; Stefano et al., 2006; Li et al., 2009; Montesinos et al., 2014). However, weaker ERD2-(X)FP signals in the tubular ER have also been recorded (Brandizzi et al., 2002; Stefano et al., 2006; Xu et al., 2012). Interestingly, the strength of the fluorescent ERD2 signal in the ER is considerably increased when p24 proteins or HDEL ligands are coexpressed (see below and Montesinos et al., 2014).

Recently, and in contrast to the findings of Montesinos et al. (2014), Silva-Alvim et al. (2018) have presented data indicating that (X)FP tagging at either the C-or N-terminus of ERD2 seems to abolish its activity in an assay measuring secretion of the artificial ligand amylase-HDEL. They therefore generated a novel ERD2b construct, consisting of a N-terminal fluorophore, followed by an extra transmembrane domain derived from the ERD2b paralog ERP1 that localizes to the ER, then ERD2b itself. This chimeric construct appeared to be functional and localizes exclusively to the Golgi, with no detectable fluorescence in the ER. Moreover, and in contrast to studies on animal cells (Lewis and Pelham, 1992; Majoul et al., 1998; Bräuer et al., 2019), even the overexpression of HDEL ligands did not change the distribution of this fluorescent construct. This is strange, and suggests that while being able to interact with HDEL ligands, this construct might not be able to undergo the conformational change which leads to COPI binding. As a result it may become trapped in the Golgi. Moreover, it is not clear whether the ERD2b construct of Silva-Alvim et al. (2018) is capable of dimerizing, or to interact with ARF1, ARF-GAP, p24 proteins and COPI subunits for sorting into COPI vesicles, which has been proposed to occur upon KDEL ligand binding (Arakel and Schwappach, 2018; Aniento et al., 2019; Bräuer et al., 2019).

Nevertheless, and despite the ongoing controversy over functionality of C- or N-tagged fluorescent ERD2 constructs in plants, an observation common to all of these studies is the predominant Golgi localization of ERD2. This poses the question: if ERD2 is supposed to cycle between the ER and the Golgi, why is ERD2 often so difficult to visualize in the ER of higher plant cells?

### The Secretory Unit and Cycling of ERD2 Between the ER and the Golgi

For our proposed model of ERD2 antero- and retrograde transport between the ER and Golgi stacks, we have made the following assumptions:

– That binding and release of HDEL-ligands to/from ERD2 in plants is also pH dependent. A pH of 7.1–7.7 for the lumen of the ER in plants has been recorded (Martinière et al., 2013), although the *cis*-Golgi pH (6.7/6.8) appears to be slightly less acidic than in mammalian cells (Shen et al., 2013; Scholl, 2018). Nevertheless, in terms of the pH response, it is not the absolute affinity for ligand in the Golgi apparatus that is important, but rather the difference between the affinities in the Golgi and the ER (Montesinos et al., 2014).
– That individual Golgi stacks and a specialized COPII export/COPI import domain of the ER network move together as a spatially defined “secretory unit” (Robinson et al., 2015). This concept is derived from confocal microscopy studies which demonstrate a coincidence of fluorescent signals for coatomer, COPII coat proteins, the recruiting GTPases Arf1 and Sar1, and the ER exit site marker Sec16 (Da Silva et al., 2004; Stefano et al., 2006; Takagi et al., 2013), as well as target membrane t-SNAREs for COPI vesicle fusion (Lerich et al., 2012). The secretory unit may be held together by direct membrane connections, antero- and retrograde tubules (see Hawes’ section in Robinson et al., 2015), but more likely by interlocking tethering factors (Osterrieder et al., 2017).
– That ERD2 in a monomeric form and without bound HDEL ligands is transported from the ER to the *cis*-Golgi in COPII vesicles. Efficient sorting of ERD2 into COPII vesicles may involve COPII-binding ER export signals (Bräuer et al., 2019). While there is no direct evidence that this occurs in plants, ER exit sites characterized by COPII proteins have been visualized by confocal imaging (Takagi et al., 2013), and COPII vesicles budding from the ER have been seen in EM images of sectioned cryo-fixed specimens in plant cells (Robinson et al., 2015).
– That dimeric/oligomeric ERD2-ligand complexes are transported in COPI vesicles back to the ER. The presence of the KDELR in COPI vesicles is well established for mammalian cells (Griffiths et al., 1994; Orci et al., 1997; Cosson et al., 2002; Stauber et al., 2006). Indeed, a recent proteomic analysis of mammalian COPI vesicles showed that KDEL receptors and p24 proteins were among the top hits of their core proteome (Adolf et al., 2019). This may also be true for plants inferred from studies where pH-dependent interactions between ERD2a, ARF1, COPI subunits, and plant p24 delta proteins have been demonstrated (Montesinos et al., 2014). It is also supported by studies on loss-of-function mutants of Arabidopsis p24 proteins, which showed increased secretion of the endogenous HDEL ligand BiP (Pastor-Cantizano et al., 2018). A putative COPI dilysine cluster such as the one described for chicken KDELR (Bräuer et al., 2019) may also be present in Arabidopsis ERD2 proteins (ERD2a/b), since both of them contain 3 lysine residues in similar positions. In addition, all of the residues that were found to be important for KDEL binding and pH-dependent conformational change of chicken KDELR (Bräuer et al., 2019) are also conserved in Arabidopsis ERD2 proteins.

### The Proposed Model

**FIGURE 1:**
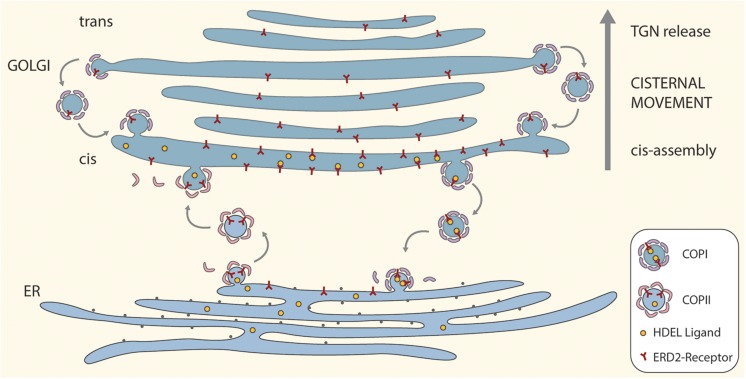
A “secretory unit” model for ERD2 cycling in higher plants. Depicted is a single Golgi stack positioned above a domain of the tubular ER network that is engaged in bidirectional trafficking of ERD2 receptors. Anterograde transport of ERD2 without attached HDEL ligands is mediated by COPII vesicles. Retrograde transport of ERD2 with bound HDEL ligands from the *cis*-Golgi is mediated by COPI vesicles. Under normal conditions ERD2 is restricted to the domain of the ER immediately underlying the *cis*-Golgi and is rapidly exported out of the ER. Under conditions where there is an excess of KDEL ligands in the Golgi, more ERD2 is transported back to the ER causing the fluorescent signal from ERD2-(X)FP to diffuse away from the ER export and import sites. Binding and dissociation of HDEL ligands to ERD2 is facilitated by the relative luminal pHs in the *cis*-Golgi and the ER. For the sake of simplicity we have not distinguished between monomeric (ER) and dimeric (Golgi) forms of ERD2 in this cartoon.

A cartoon incorporating the salient features of the proposed ERD2 cycling model is given as Figure 1. For the sake of simplicity and clarity, the tethering factors between the ER and the *cis*-Golgi have been omitted, as also putative tubular connections. So too have possible pre-*cis-*cisternal compartments e.g., the so-called GECCO (Golgi-entry core compartment, Ito et al., 2018) lying between the ER and the Golgi stack have not been drawn. While more than one *cis*-cisterna may be involved in cargo recognition and receptor-ligand retrieval, these events have been drawn only for the first *cis*-cisterna. This is a realistic supposition since some ER resident proteins e.g., calreticulin (Crofts et al., 1999) which has an HDEL motif, or the KDEL-tagged version of the trimeric vacuolar storage protein phaseolin (Frigerio et al., 2001), have N-linked oligosaccharide chains of the high-mannose type. These oligosaccharides are structurally available for processing, but remain unprocessed indicating that these proteins do not enter median or *trans* cisternae and are effectively retrieved from the *cis*-Golgi.

The model also incorporates an efficient recovery system for ERD2 from late Golgi cisternae. Such a mechanism is required to counteract the loss of ERD2 carried upward through the Golgi stack as a consequence of continual *cis*-cisternal assembly and ongoing cisternal displacement through the stack. It is supported by recent observations on the COPI-mediated retrieval of glycosyl transferases in the mammalian Golgi apparatus (Liu et al., 2018), and is an integral feature of the cisternal maturation model for Golgi function (Donohoe et al., 2013; Ito et al., 2014).

In terms of membrane equilibrium in the early secretory pathway, the amount of vesicle membrane being exported via COPII to the Golgi should be the same as the amount of vesicle membrane being returned to the ER via COPI. So, under steady-state conditions it is unlikely that fewer COPI vesicles are being released from the Golgi than COPII vesicles from the ER. Thus higher concentrations of ERD2 in the Golgi cannot be a result of a reduced retrograde vesicle traffic.

A possible scenario for ERD2 function is as follows: ligand-free monomeric ERD2 in the ER is constitutively and continually exported via COPII to the Golgi. In the Golgi, at acidic pH, ERD2 binds HDEL-ligands leading to dimerization and a conformational change in ERD2 which exposes lysine residues to generate a COPI binding motif. This means that the receptor will only enter COPI vesicles (and be retrieved to the ER) after ligand binding. Once in the ER, and due to the neutral pH in this compartment, ERD2 dissociates from HDEL ligands and can be exported again to the Golgi. ERD2 may, however, temporarily accumulate in the Golgi when the concentration of HDEL ligands is low.

The model also shows, that, under normal conditions, ERD2 in the ER is only transitorily present and is rapidly cleared from this compartment. When present, it is restricted to a disc-like domain immediately subjacent to the first *cis*-Golgi cisterna, where COPI vesicles fuse and COPII vesicles exit. A common feature of the steady state distribution of KDELR in mammals and ERD2 in plant cells is that receptor labeling of the ER is very low. Only when the receptor or HDEL-ligands are overexpressed does KDELR/ERD2 diffuse out into the body of the tubular ER system (Lewis and Pelham, 1992; Tang et al., 1993; Montesinos et al., 2014; Bräuer et al., 2019). This is also the case upon increased function of p24 proteins, which presumably facilitate sorting of ERD2 into nascent COPI vesicles (Montesinos et al., 2014; Pastor-Cantizano et al., 2016; Aniento et al., 2019).

## Discussion

With this model the predominant location of ERD2 in the Golgi, with little ERD2 visible in the ER, can be explained on the basis of a classical pH-dependent HDEL-ligand binding and release mechanism, employing COPI-/COPII-vesicles as transport carriers, but also taking into account the unique structural features of the early secretory system of higher plants. It explains why ERD2 molecules are difficult to visualize in the ER under steady-state conditions, because they are situated in a restricted domain in close proximity to the *cis*-Golgi and cannot therefore be clearly differentiated from the greater mass of ERD2 in the *cis*-Golgi when the stacks are viewed from above or below. This problem might, however, be overcome by high resolution side-on imaging, and possibly by immunogold electron microscopy.

In order to explain the extreme difference in ERD2 concentrations in the ER and Golgi, Silva-Alvim et al. (2018) have postulated that anterograde ER to Golgi transport of ERD2 may be extremely fast. Could this be achieved by direct tubular connections between the ER and the Golgi? Such structures have been discussed in the plant literature for quite some time (Hawes et al., 2008; Hawes, 2012; Hawes section in Robinson et al., 2015), but remain controversial. In contrast, in mammalian cells there have recently been convincing reports for tubular ER-Golgi connections. Some of these are formed to transport large molecules e.g., procollagen, that cannot be packaged into a small (<100 nm diam) COPII vesicles (Raote and Malhotra, 2019). Interestingly, although collagen is not synthesized by plants, human trimeric pocollagen has been sucessfully expressed ectopically in transgenic plants, and is in fact successfully targeted to the vacuole when tagged with a vacuolar sorting signal suggesting that it travels through the Golgi (Stein et al., 2009). Unfortunately, this study lacked EM observations so there is no information on the nature of the ER-Golgi transport vector. Moreover, although collagen fibrils could be formed from vacuolar extracts this required heating to 37°C for 1h so that large procollagen aggregates may not be present in the ER/Golgi lumen. Transgenic cases such as this may induce the formation of COPII-coated tubules, but do not necessarily reflect the wild type situation. We can conclude that not only might the ER-Golgi transport vector be dependent on the type of secretory cargo, but may also, as discussed by Brandizzi (2018), reflect the spatial organization of the ER and the Golgi apparatus in plants which can differ between various cell types. So a generalization for or against tubules may not be possible to make at this time.

Much thinner tubules emanating from the Golgi in HeLa cells have recently been described by Bottanelli et al. (2017) through the use of ARF1 endogenously tagged with Halo. Anterograde tubules, with budding clathrin coated vesicles, were seen to form at the TGN, but not at the ER. Retrograde Golgi tubules were also observed and KDELR were visualized in them. However, this represented only a small percentage of the total receptor population, the bulk of KDELR presumably being transported via COPI to the ER (Béthune and Wieland, 2018; Adolf et al., 2019). It was also calculated that these tubules could only account for less than 10% of the total membrane transported retrogradely out of the Golgi. Even though these tubules may be partially coated with COPI/COPII proteins, it is not immediately apparent how movement of a transmembrane receptor in the plane of the tubule membrane could be faster in terms of traversing the ER-Golgi interface than being transported in a vesicle. It is also difficult to see how a pH gradient between ER and *cis*-Golgi can be maintained when the two compartments have lumenal continuity. Thus, it is unlikely that tubules are a contributing factor to the extreme assymetric distribution of ERD2 in the early secretory pathway of higher plant cells. Alternatively, ER to Golgi transport of ERD2 may occur via standard COPII vesicles. In this respect, ERD2 may contain COPII-binding ER export signals, as suggested for acidic residues present in the cytosolic domains of chicken KDEL receptor (Bräuer et al., 2019), which are conserved in Arabidopsis ERD2 proteins. Perhaps this may also be achieved by a dileucine motif in its cytoplasmic tail (Silva-Alvim et al., 2019).

The paper of Silva-Alvim et al. (2018) has raised doubts as to the universality of COPI-mediated retrograde transport of ERD2-HDEL ligands from the Golgi apparatus. This is quite a singular paper in the large body of literature on ERD2/KDEL receptors. As indicated above, there remain doubts as to the fidelity of the results obtained with their novel fluorescent ERD2b construct. However, one notes that in their recent study on chicken KDELR, Bräuer et al. (2019) also tagged their receptor with GFP at the C-terminus, but added a 20 aa linker between the GFP and the C-terminus. Presumably, this prevents any interference with sorting signals for COPI binding, and should be a useful strategy to follow in the future. Indeed, this fusion protein was still able to redistribute to the ER upon ligand binding unless residues involved in ligand or COPI binding were mutated (Bräuer et al., 2019).

Ideally, key features of ERD2 trafficking should be preserved in any fluorescent reporter, including its ability to dimerize, which has been shown both in mammals (Aoe et al., 1997; Majoul et al., 2001, 2002) and in plants (Xu and Liu, 2012), as well as its ability to interact with components involved in COPI vesicle formation, including p24 proteins and COPI subunits themselves, as shown both in mammals (Majoul et al., 2001, 2002) and in plants (Montesinos et al., 2014). On the other hand, it would seem necessary to investigate the subcellular localization of endogenous ERD2 proteins by immunogold electron microscopy or immunofluorescence. In addition, by using a plant stably expressing an HDEL-cargo under the control of an inducible promoter, one might be able to detect a partial redistribution of ERD2 following synthesis of HDEL cargo.

## Author Contributions

Both authors listed have made a substantial, direct and intellectual contribution to the work, and approved it for publication.

## Conflict of Interest

The authors declare that the research was conducted in the absence of any commercial or financial relationships that could be construed as a potential conflict of interest.
